# Differential metabolism-associated gene expression of duck pancreatic cells in response to two strains of duck hepatitis A virus type 1

**DOI:** 10.1007/s00705-021-05199-4

**Published:** 2021-09-05

**Authors:** Zhen Chen, Shao-hua Shi, Yu Huang, Cui-qin Huang, Rong-chang Liu, Long-fei Cheng, Guang-hua Fu, Hong-mei Chen, Chun-he Wan, Qiu-ling Fu

**Affiliations:** 1 Institute of Animal Husbandry and Veterinary Medicine, Fujian Academy of Agricultural Sciences, Fuzhou, 350013 Fujian People’s Republic of China; 2 Fujian Provincial Key Laboratory for Avian Diseases Control and Prevention, Fuzhou, 350013 Fujian People’s Republic of China; 3 Fujian Animal Diseases Control Technology Development Center, Fuzhou, 350013 Fujian People’s Republic of China; 4 College of Life Sciences, Longyan University, Longyan, 364012 Fujian People’s Republic of China

## Abstract

Several outbreaks of duck hepatitis A virus type 1 (DHAV-1), which were characterized by yellow coloration and hemorrhage in pancreatic tissues, have occurred in China. The causative agent is called pancreatitis-associated DHAV-1. The mechanisms involved in pancreatitis-associated DHAV-1 infection are still unclear. Transcriptome analysis of duck pancreas infected with classical-type DHAV-1 and pancreatitis-associated DHAV-1 was carried out. Deep sequencing with Illumina-Solexa resulted in a total of 53.9 Gb of clean data from the cDNA library of the pancreas, and a total of 29,597 unigenes with an average length of 993.43 bp were generated by *de novo* sequence assembly. The expression levels of D-3-phosphoglycerate dehydrogenase, phosphoserine aminotransferase, and phosphoserine phosphatase, which are involved in glycine, serine, and threonine metabolism pathways, were significantly downregulated in ducks infected with pancreatitis-associated DHAV-1 compared with those infected with classical-type DHAV-1. These findings provide information regarding differences in expression levels of metabolism-associated genes between ducks infected with pancreatitis-associated DHAV-1 and those infected with classical-type DHAV-1, indicating that intensive metabolism disorders may contribute to the different phenotypes of DHAV-1-infection.

## Introduction

Duck viral hepatitis (DVH) is a highly fatal contagious disease of ducklings that is characterized by clinical signs of opisthotonos and by hemorrhagic lesions of the liver [1]. This causes mortality rate is approximately 95% in ducklings within a week. The causative agents of DVH include duck hepatitis virus type 1 (DHV-1), DHV-2, and DHV-3. Recently, DHV-1, along with two other types, was classified as a member of the species *Avihepatovirus* A of the genus *Avihepatovirus* in the family *Picornaviridae* and was renamed as duck hepatitis A virus (DHAV) [2]. DHAV was furtherly divided into three genotypes: DHAV-1, DHAV-2, and DHAV-3.

DHAV-1 is distributed worldwide, and DHAV-2 has been isolated only in Taiwan to date [3, 4]. The presence of DHAV-3 was mainly reported in Korea and China [5–8]. Generally, DHAV-1 causes lesions in the liver that are typical of DVH. However, Guérin et al. reported in 2005 that several DHV infections of young Muscovy ducklings resulted in nervous symptoms and pancreatitis [9]. Several outbreaks of DHAV-1, which were characterized by yellow coloration and hemorrhage in pancreatic tissues, occurred in China in 2011 [10]. The causative agent was subsequently named "pancreatitis-associated DHAV-1". Genome sequencing indicated a variation rate of 3.4-6.5% in the genome of pancreatitis-associated DHAV-1 compared with that of classical-type DHAV-1 [11]. The antigenic association between pancreatitis-associated DHAV-1 and the classical-type DHAV-1 indicated large variation [12]. The comparative pathogenicity of pancreatitis-associated DHAV-1 and the classical type DHAV-1 in the ducklings of different species has been well documented [13, 14]. However, the mechanisms involved in infection by pancreatitis-associated DHAV-1 are still unclear. The host response to pancreatitis-associated DHAV-1 infection requires experimental investigation.

The host-pathogen interaction is a complex and dynamic biological system with its outcome depending on the ability of the microbial pathogen to establish infection and the ability of the host to control infection [15]. Transcriptomics is the analysis of genome-wide RNA expression and is an available approach to characterize host and pathogen processes in infectious diseases. This method also provides a clear understanding of host defense mechanisms during viral infection. To date, data on the transcriptome profile of the duck pancreatic tissue and its response to DHAV-infection at the transcriptome level are lacking. Moreover, differences between the host responses to classical-type DHAV-1 and pancreatitis-associated DHAV-1 have not been reported to date.

In the present study, transcriptome sequencing was applied to determine the transcriptome pattern of duck pancreatic tissue infected with classical-type DHAV-1 and pancreatitis-associated DHAV-1. Dramatic differences were observed in the expression of genes involved in glycine, serine, and threonine metabolism pathway, indicating that intensive metabolism disorders may contribute to different phenotypes of DHAV-1-infection.

## Materials and methods

### Animal experiment

One-day-old Muscovy ducklings (*Cairina moschata*) were purchased from a commercial hatchery farm. Each duckling had free access to food and water and shared the same environmental conditions at 35 ℃. The temperature was decreased by 1 ℃ every day. Light was continuously available during the animal experiments. Serum samples and cloacal swabs were collected from the ducklings prior to viral challenge. The presence of antibody and antigen was determined using indirect enzyme-linked immunosorbent assay (ELISA) and RT-PCR methods [10], respectively, in order to confirm that the animals were negative for classical-type DHAV-1 and pancreatitis-associated DHAV-1. Four days later, 60 5-day-old healthy Muscovy ducklings that were negative for classical-type DHAV-1 and pancreatitis-associated DHAV-1 antigens and antibodies, were divided randomly into three groups (20 in each group). The ducklings in each group were challenged intramuscularly with 10^5.0^ ELD_50_ (50% egg lethal dose) of classical-type DHAV-1 strain FZ86 and pancreatitis-associated DHAV-1 GD1206 strain in 200 µl of phosphate-buffered saline (PBS) or mock-infected with PBS. All DHAV-1 isolates were obtained from naturally infected ducks in China. DHAV-1 strain FZ86 was isolated in our laboratory in Fujian, China, in 1986 and was characterized by causing liver hemorrhage in infected ducklings. DHAV-1 strain GD1206 was isolated in our laboratory in Guangdong, China, in 2012 and was characterized by causing pancreatitis in infected ducklings [10, 13]. Each group of ducklings was housed in separate isolators (Suzhou Fung's Laboratory Animal Equipment Co., Ltd.) with free access to food and water throughout the experimental period. Each group of ducklings shared the same environmental conditions at an experimental temperature of 31 ℃. The temperature was reduced by 1 ℃ every day until reaching 25 ℃. Light was continuously available during the animal experiments. Clinical signs were monitored daily.

At 24 h post-inoculation, six ducklings in each group were selected randomly and administered sodium pentobarbital (40 mg/kg body weight; Sigma-Aldrich, USA) intravenously, and subsequently, the animals were humanly sacrificed under narcosis [16]. Pancreatic samples were harvested for transcriptional analysis. Concomitantly, the pancreatic samples were also tested by RT-PCR in order to confirm classical-type DHAV-1 or pancreatitis-associated DHAV-1 infection. The remaining 14 ducklings of each group were observed for three days. At the end of the observation period, the ducklings were administered sodium pentobarbital (40 mg/kg body weight; Sigma-Aldrich, USA) intravenously and subsequently humanly sacrificed under narcosis [16]. The gross lesions were observed.

### Host gene expression associated with pancreatitis-associated DHAV-1 infection

#### RNA extraction, library construction, and sequencing

Total RNA was extracted using RNAiso Plus Total RNA Extraction Reagent (TaKaRa, Shiga, Japan) according to the manufacturer’s instructions. RNA degradation and contamination were monitored by electrophoresis in 1.0% agarose gels. RNA purity was confirmed using a Nano Photometer® spectrophotometer (IMPLEN, CA, USA). The RNA concentration was measured using a Qubit® RNA Assay Kit in a Qubit®2.0 Fluorometer (Life Technologies, CA, USA). RNA integrity was assessed using an RNA Nano 6000 Assay Kit and an Agilent Bioanalyzer 2100 system (Agilent Technologies, CA, USA) and subsequently employed as a template for the synthesis of double-stranded cDNA. Sequencing libraries were generated using an NEB Next®Ultra™ RNA Library Prep Kit for Illumina^®^ (NEB, CA, USA) following the manufacturer’s recommendations. Finally, index codes were added to the attribute sequences of each sample. The clustering of the index-coded samples was performed on a cBot Cluster Generation System using a TruSeq PE Cluster Kit v3-cBot-HS (Illumina) according to the manufacturer’s instructions. Following cluster generation, the library preparations were sequenced on an Illumina Hiseq 4000 platform, and paired-end reads were generated. Raw reads were filtered and assembled using Trinity software, and a transcript library was obtained. The quantitative relationship between the transcript and the genes was analyzed. Using Bowtie and RSEM software, we calculated expression levels using paired reads in the transcript library and determining the count value. The count of expression was normalized according to the length of the transcript. The RNA-seq data from this study were submitted to the NCBI Sequence Read Archive (SRA) (http://www.ncbi.nlm.nih.gov/sra/).

### Transcriptome data analysis

Differential expression analysis of the three groups was performed using the DESeq R package (1.10.1). DESeq provided statistical routine methods for determining differential expression in digital gene expression data using a model based on a negative binomial distribution. The resulting *P*-values were adjusted using the method of Benjamini and Hochberg for controlling the false-discovery rate. Genes with an adjusted *P*-value of <0.05, identified using DESeq, were identified as differentially expressed genes.

### GO enrichment analysis and KEGG pathway enrichment analysis

Gene Ontology (GO) enrichment analysis of the differentially expressed genes (DEGs) was implemented by the topGO R package-based Kolmogorov–Smirnov test. The GO terms with corrected *P*-value less than 0.05 were considered significantly enriched in the differentially expressed genes.

The Kyoto Encyclopedia of Genes and Genomes (KEGG) is a database resource for understanding high-level functions and utilities of biological systems, such as cells, organisms, and ecosystems. Specific molecular-level information is collected from large-scale molecular datasets generated by genome sequencing and other high-throughput experimental technologies (http://www.genome.jp/kegg/) [17]. The KOBAS software was used to assess the statistical enrichment of differentially expressed genes in the KEGG pathways [18]. KEGG terms with a corrected *P*-value (Q-value) less than 0.05 were considered significantly enriched in the differentially expressed genes.

### Real-time RT-PCR

The expression levels of the differentially expressed genes were also assessed by real-time RT-PCR in order to confirm the sequencing data. The individual primer sequences of the 10 target genes and of the internal reference gene (GAPDH) [19] are listed in Table 1. Following reverse transcription, a SYBR®Fast qPCR Mix (Takara, Beijing) and a Mastercycler ep realplex (Eppendorf) were used according to the manufacturers’ instructions. The reaction conditions were as follows: 95°C for 1 min, followed by 40 cycles of 95°C for 10 s and 60°C for 40 s. A final melt curve analysis was completed. The relative mRNA levels were compared to those of the mock-infected ducks and the mean values were calculated using the threshold cycle 2^-△△CT^ method [20]. Differences between experimental groups were evaluated using Student’s *t*-test with a two-tailed analysis. *P*-values less than 0.05 were considered significant.

**Table 1:** Primer sequences of differentially expressed genes for real-time RT-PCR analysis

Primer name	Sequence (5′- 3′)	Size (bp)
GAPDH [19]	F-ATGTTCGTGATGGGTGTGA R-CTGTCTTCGTGTGTGGCTGT	176
GNMT-like	F-CAGCTGGCTCCTGGCGCTGC	183
	R-GGCTCCTCCTTGCGCCGCTC	
GCAT	F-GCTCCGGCGGCGGCTGGAGG	171
	R-GGGTGGCTCGACAGCCCCAGGTA	
CBS	F-GTACCTCCTGTGCCAGCTGTAGCCA	170
	R-GTGGGAGTAGCGTGGAGACTGAAAA	
PHGDH	F-CCAAGACCCCGGGCTCGCAGCCACC	177
	R-CGTGGGGCTCCCGACCAAGAGGCTG	
SERCA	F-ACGGCCTTCGTCGAGCCCTTCGTCA	186
	R-GGGCACGAGGTCCCTGGCCTTGATG	
PLCγ	F-GTTTTCTGTGATCAGAAATACAC	204
	R-TATTTAAAACCAGTTGTCCTAATTT	
TLR2	F-CTCTTCTTCACGAGGCCACTCCAGG	183
	R-CAGAGCGAGTGGTGCAAGTACGAGC	
TLR4	F-TCACCAGCCTACAAGACCTGCAAGA	184
	R-GGAGGAGGTGGACGGAGGCACTGTA	
TLR7	F-CATGCACTCCCCACTGGAAGTCCCT	150
	R-CTTCCTGGCAGCCTGTGTGCTAAAG	
IFNα	F-GCCCAGATGCGGGACTGTCCACCTG	167
	R-ACACGGCTGCACGATGGGAATCTCC	

## Results

### Gross lesions in ducklings infected with classical-type DHAV-1 and pancreatitis-associated DHAV-1

**Fig. 1:**
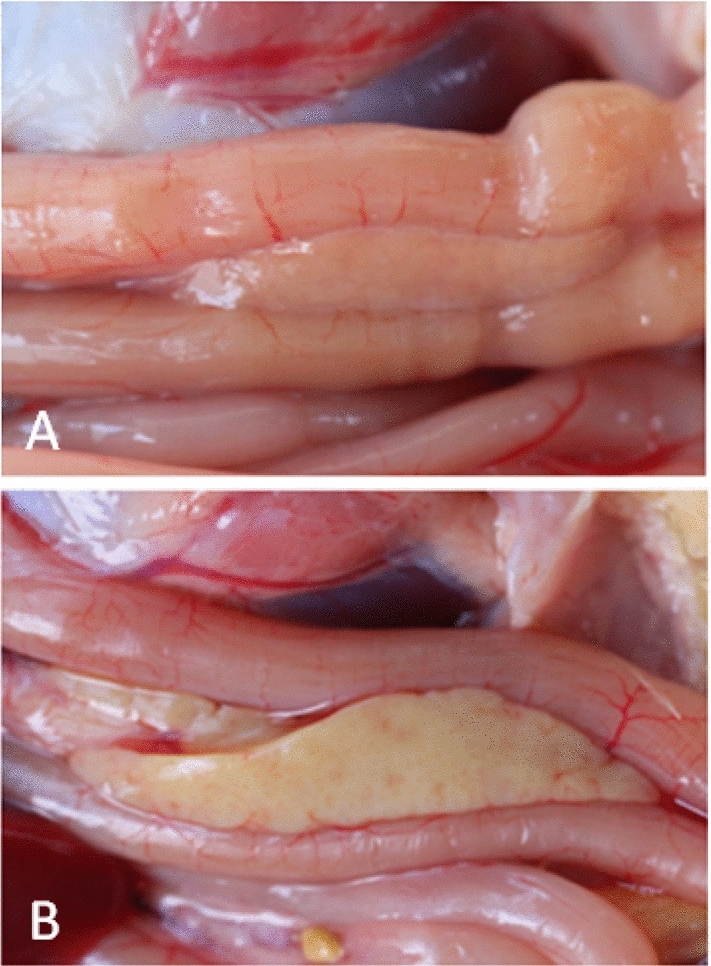
Gross lesions in ducklings infected with classical-type DHAV-1 (A) and pancreatitis-associated DHAV-1- (B)

Infection experiments were conducted using the pancreatitis-associated DHAV-1 strain GD1206 and the classical-type DHAV-1 strain FZ86 at a dose of 10^5.0^ ELD_50_ per duck. The ducks infected with the classical-type DHAV-1 FZ86 strain showed clinical signs of depression, lethargy, and opisthotonos. Major lesions that were typically enlarged with petechial and ecchymotic hemorrhages in the liver and kidney were observed during necropsy. No visible lesions were noted in the pancreas. In contrast to these observations, the ducklings infected with the pancreatitis-associated DHAV-1 GD1206 strain demonstrated no typical signs during the course of infection. The major affected organ was the pancreas, which exhibited lesions of yellowing or hemorrhage. No hemorrhagic lesions were noted in the liver of the pancreatitis-associated DHAV-1-infected ducklings (Fig. 1). No gross lesions were observed in the mock-infected control group. The pancreatic samples tested positive for the respective DHAV-1 type by reverse transcription polymerase chain reaction (RT-PCR).

### RNA-seq and assembly of transcriptome data from pancreas tissues of infected ducklings

Using Illumina-Solexa deep sequencing, a total of 183 M raw reads were obtained from a cDNA library derived from pancreatic tissues. The removal of low-quality reads (i.e., reads containing only adaptors and empty reads) resulted in the identification of clean reads with 53.9 Gb of clean data. *De novo* sequence assembly resulted in a total of 29,597 unigenes with an average length of 993.43 bp. BLAST and ORF analyses indicated that only 9,387 unigenes matched known genes. The sequencing data from GD1206- and FZ86-infected ducks were submitted to the NCBI database (accession numbers: SRR7239978, SRR7239979, SRR7239984, SRR7239985, SRR7239988, and SRR7239989).

**Fig. 2:**
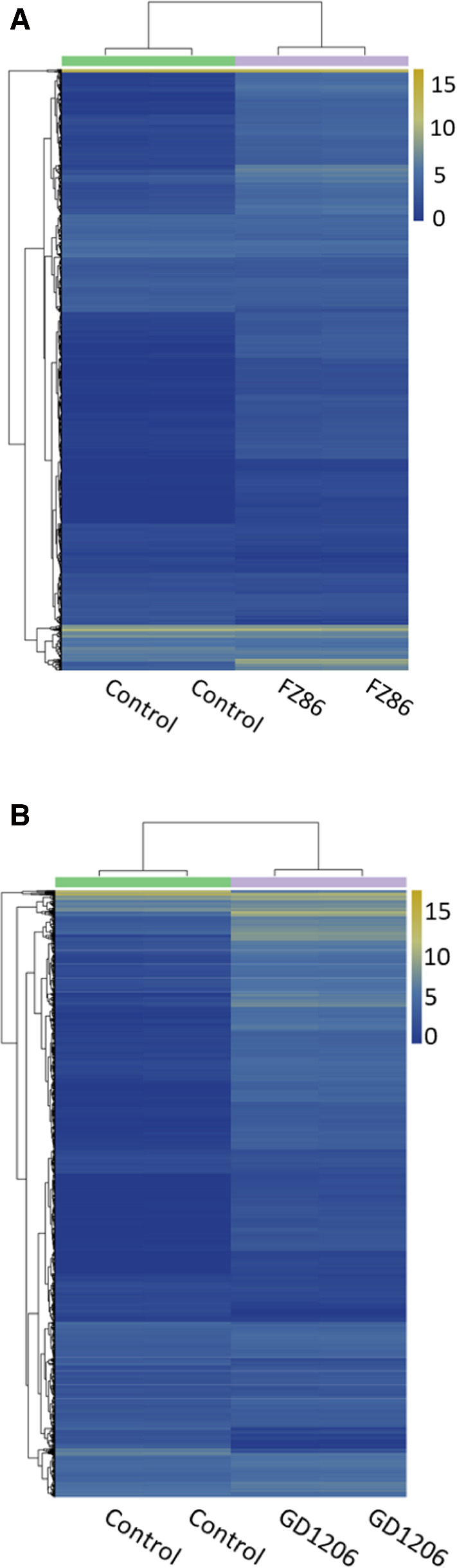
Heat map analysis for classification of gene expression patterns in classical-type DHAV-1 strain FZ86 infection (A) and pancreatitis-associated DHAV-1 strain GD1206 infection (B). Genes with similar expression patterns were clustered, as shown in the heat maps. The intensity of color indicates gene expression levels normalized according to Log_2_FPKM value

**Fig. 3:**
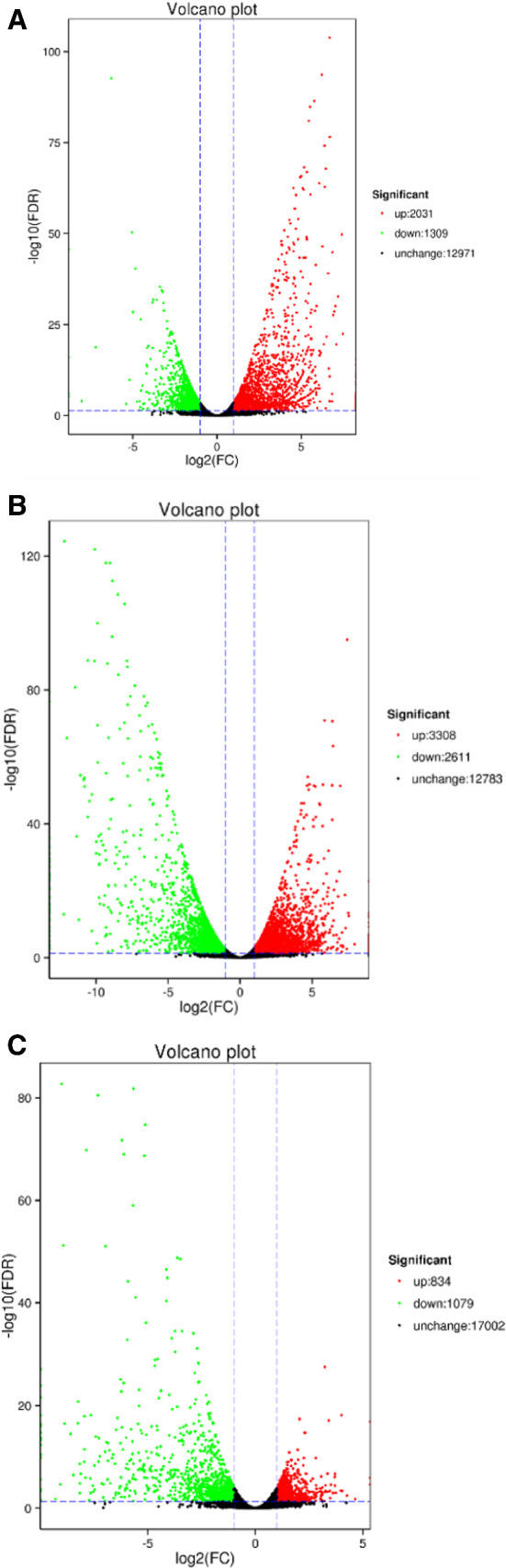
Volcano plots of differentially expressed genes in the pancreas of ducklings in classical-type DHAV-1 vs. control (A), pancreatitis-associated DHAV-1 vs. control (B), and pancreatitis-associated DHAV-1 vs. classical-type DHAV-1 (C). Red points represent upregulated genes, green points represent downregulated genes, and blue points represent genes with no significant difference

Heat maps were used to identify the top differentially expressed genes (DEGs) and to classify gene expression profiles in DHAV-1-infected tissues (Fig. 2). In order to obtain a global view of the differences in duck gene expression among the different experimental groups, three paired comparisons (classical-type DHAV-1 vs. control, pancreatitis-associated DHAV-1 vs. control, pancreatitis-associated vs. classical-type DHAV-1) were performed. RNA-seq analysis identified 3,340 and 5,919 genes that were expressed at significantly different levels in classical-type-DHAV-1- and pancreatitis-associated-DHAV-1-infected animals, respectively, than in the control group (*P* < 0.05). Classical-type DHAV-1 infection contributed to the differential expression of 2,031 genes that were upregulated and 1,309 genes that were downregulated in the pancreatic tissues of the infected ducklings compared to the controls. Moreover, 3,308 genes were upregulated and 2,611 genes were downregulated in the pancreatitis-associated-DHAV-1-infected animals. A total of 1,913 genes were differentially expressed in pancreatitis-associated-DHAV-1-infected animals compared with the classical-type DHAV-1 group. Specifically, 834 genes were downregulated and 1,079 genes were upregulated (Fig. 3).

### Functional analysis of the transcriptome data derived from the pancreatic tissues of different DHAV-1-infected animals

We performed GO enrichment analysis by functional annotation clustering of DEGs and annotated DEGs into three groups, including biological processes (BP), cellular components (CC), and molecular function (MF).

**Fig. 4:**
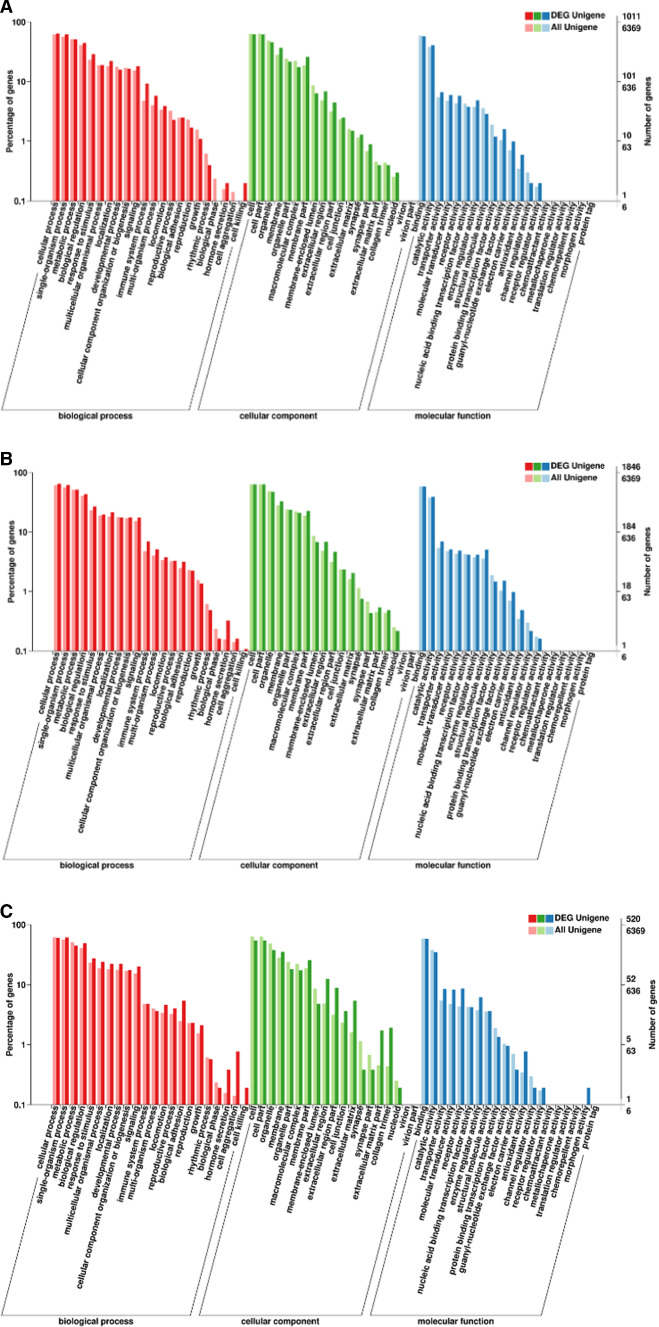
GO classification of differentially expressed genes in the pancreas of ducklings in classical-type DHAV-1 vs. control (A), and pancreatitis-associated DHAV-1 vs. control (B), pancreatitis-associated DHAV-1 vs. classical type DHAV-1 (C). Annotation statistics of differentially expressed genes in the secondary node of GO. The horizontal axis shows secondary nodes of three categories in GO. The left side of the vertical axis displays the percentage of annotated genes versus the total gene number, and the right side shows the number of genes.

The putative functions of the unigenes in the libraries derived from the classical-type-DHAV-infected ducklings were analyzed using GO. The analysis of the GO categories of the classical-type DHAV-1 group indicated that the differentially expressed genes were mapped to 61 categories of BP, CC, and MF (Fig. 4A). The categories of BP included genes that are mainly involved in the positive regulation of interleukin-12 production, myeloid leukocyte activation, and T cell proliferation. The majority of the corresponding genes in the CC categories affect the external side of plasma membrane, the oligosaccharyltransferase complex, and the integral component of the membrane. The majority of the corresponding genes in the MF categories were involved in non-membrane-spanning protein tyrosine kinase activity, antioxidant activity, and calcium-dependent phospholipid binding.

The putative functions of the unigenes in the pancreatic libraries affected by pancreatitis-associated DHAV-1 infection were analyzed using GO. The analysis of the GO categories indicated that the differentially expressed genes were mapped to 61 categories, including BP, CC, and MF (Fig. 4B). The majority of the corresponding genes in the BP categories were involved in cholesterol efflux, positive regulation of the cell cycle, and translation. The categories of the CC included genes that were mainly associated with the cytosolic large ribosomal subunit, extracellular space, and ribosome. The majority of the genes of the MF categories included genes involved in the structural constituents of the ribosome, cytokine activity, and NADH dehydrogenase (ubiquinone) activity.

The putative functions of the unigenes in the pancreatic libraries of the pancreatitis-associated-DHAV-1-infected ducklings were compared with those of the classical-type-DHAV-1-infected ducklings and were analyzed using GO. The analysis of the GO categories indicated mapping of differentially expressed genes to 61 categories of BP, CC, and MF (Fig. 4C). The BP categories mainly included genes that were involved in the oxidation-reduction process, negative regulation of the apoptotic process, and myeloid leukocyte activation. The CC categories mainly included genes that affect the extracellular space, protein-extracellular matrix, and ribosome. The majority of the corresponding genes in the MF categories are associated with antioxidant activity, G-protein-coupled peptide receptor activity, and structural constituents of the ribosome.

### Pathway analysis of DEGs based on KEGG after infection with different DHAV-1 types

**Fig. 5:**
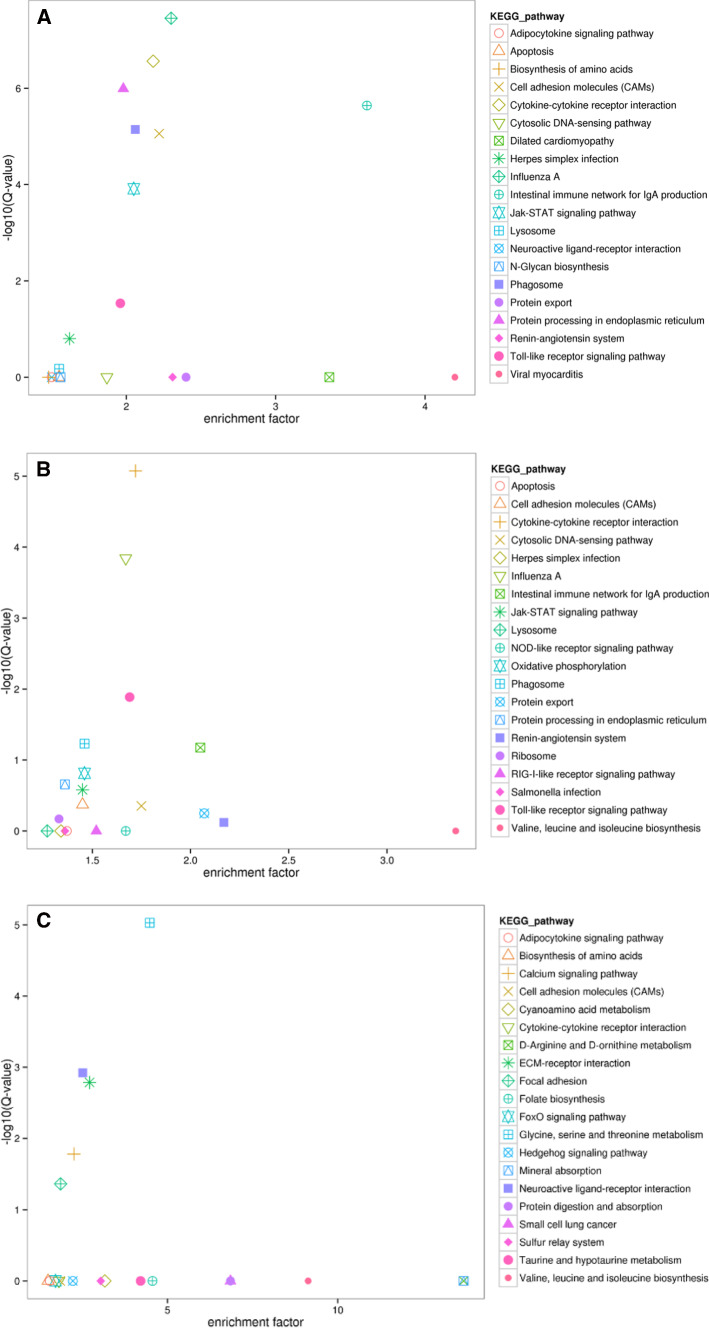
Top 20 KEGG pathways enriched in the pancreas of ducklings in classical-type DHAV-1 vs. control (A), pancreatitis-associated DHAV-1 vs. control (B), and pancreatitis-associated DHAV-1 vs. classical type DHAV-1 (C)

The KEGG database was used to analyze specific pathways in order to further define DEG function in duckling pancreatic tissue following infections with different DHAV-1 types. The top 20 enrichment KEGG pathways are listed in Figure 5 according to their Q-value (Q < 0.05) (Table 2).

**Table 2:** Signal pathway enrichment evaluated by KEGG in the pancreas of ducklings in classical-type DHAV-1 vs. control, pancreatitis-associated DHAV-1 vs. control, and pancreatitis-associated DHAV-1 vs. classical-type DHAV-1

Group	Pathway	DEGs in pathway	All genes in pathway	*P*-value	*Q*-value
Classical-type DHAV-1 vs. control	Influenza A	55	134	1.58E-10	3.48E-08
	Cytokine-cytokine receptor interaction	56	144	1.24E-09	2.73E-07
	Protein processing in endoplasmic reticulum	66	187	4.65E-09	1.02E-06
	Intestinal immune network for IgA production	20	31	1.03E-08	2.27E-06
	Phagosome	53	144	3.26E-08	7.18E-06
	Cell adhesion molecules (CAMs)	44	111	3.98E-08	8.76E-06
	JAK-STAT signaling pathway	44	120	5.58E-07	0.000123
	Toll-like receptor signaling pathway	29	83	0.000132203	0.029085
Pancreatitis-associated DHAV-1 vs. control	Cytokine-cytokine receptor interaction	74	144	3.43E-08	8.44E-06
	Influenza A	67	134	5.84E-07	0.000144
	Toll-like receptor signaling pathway	42	83	5.28E-05	0.012986
Pancreatitis-associated DHAV-1 vs. classical type DHAV-1	Glycine, serine and threonine metabolism	17	52	6.74E-08	9.37E-06
	Neuroactive ligand-receptor interaction	26	142	8.65E-06	0.001202
	ECM-receptor interaction	22	111	1.18E-05	0.001637
	Calcium signaling pathway	24	146	0.000118608	0.016487
	Focal adhesion	33	243	0.000311296	0.04327

A total of six functional categories were identified that play important roles in the classical-type DHAV-1 FZ86 and pancreatitis-associated DHAV-1 GD1206 infections. These categories were mainly associated with the immune system, including the Toll-like receptor signaling pathway. However, significant KEGG enrichment in the pancreatitis-associated DHAV-1 group was also involved in metabolism, including the glycine, serine, and threonine metabolism pathways.

### Verification of DEG identification by real-time RT-PCR

In order to verify the differential gene expression levels obtained from the transcriptome sequencing data, we analyzed the expression levels of 10 genes involved in immune and metabolism-associated functions. These genes were also involved in host immune defense responses and metabolic function noted in the DHAV-1 infection groups. The genes examined were as follows: GNMT-like, GCAT, CBS, PHGDH, SERCA, PLCγ, TLR2, TLR4, TLR7, and IFNα. They were differentially expressed compared with the control (*P* < 0.05), indicating the reliability of the transcriptome sequencing data (Table 3).

**Table 3:** Verification by real-time RT-PCR for some differentially expressed genes

Gene	Abbreviation	Transcriptomics fold change (Log_2_FC)		Real-time RT-PCR fold change (2^-∆∆CT^)	
		Classical type DHAV-1/control	Pancreatitis-associated DHAV-1/control	Classical type DHAV-1/control	Pancreatitis-associated DHAV-1/control
Glycine N-methyltransferase-like	GNMT-like	-2.08	-5.72	0.210	0.01
Glycine C-acetyltransferase	GCAT	-2.11	-4.80	0.20	0.04
Cystathionine beta-synthase	CBS	-1.87	-5.79	0.25	0.03
Phosphoglycerate dehydrogenase	PHGDH	-2.49	-5.24	0.18	0.02
Sarcoplasmic/endoplasmic reticulum calcium ATPase	SERCA	1.05	1.05	2.46	4.48
1-phosphatidylinositol 4,5-bisphosphate phosphodiesterase gamma	PLCγ	1.58	1.37	4.96	2.95
Toll-like receptor 2	TLR2	2.93	3.41	8.69	12.30
Toll-like receptor 4	TLR4	5.27	5.69	38.05	46.85
Toll-like receptor 7	TLR7	5.81	5.36	45.57	30.27
Interferon alpha	IFNα	2.23	2.62	3.89	8.06

## Discussion

In the present study, ducklings that were inoculated with the classical-type DHAV-1 strain FZ86 developed massive haemorrhages on the liver surface. The ducklings that were inoculated with pancreatitis-associated DHAV-1 strain GD1206 exhibited loss of appetite, lying prone, diarrhea, and depression. The gross lesions of the yellowed or hemorrhagic pancreatitis were observed in the pancreatitis-associated-DHAV-1-infected ducklings, but no consistent nervous disorders were observed in the present study, which is not in line with the observations of Guérin et al. [9]. Cha et al. reported that a DHAV-3 strain induced only liver discoloration without hemorrhagic mottling, lymphocyte infiltration, or bile duct hyperplasia, as determined by histology of the lesions [21]. These studies reveal the diversity of the pathogenic effects of DHAV infection.

Transcriptome analysis is a promising tool that can provide a comprehensive understanding of the molecular mechanisms involved in specific biological processes and diseases [22]. Previous studies have described the transcriptome profiles of DHAV, reovirus, and DHBV infections in ducks [19, 23–25]. In the present study, transcriptome sequencing was employed to explore and compare the gene expression patterns of infection with different DHAV-1 types in duck pancreatic tissues, with the aim of comparing different molecular events during pancreatitis-associated DHAV-1 infection.

Analysis of the clean reads with BLAST and ORF resulted in a total of 9,387 matched known genes. The DEGs were annotated and categorized by GO and KEGG signaling pathway analyses, which demonstrated that the majority of these genes in the classical-type DHAV-1and pancreatitis-associated DHAV-1 infection groups were classified in the immune system and metabolism categories. Comparisons of the transcriptomes of classical-type-DHAV-1-infected and the control ducklings indicated that classical-type DHAV-1 infection caused downregulation of genes associated with metabolic pathways and inhibition of the metabolism of the host cell. In addition, upregulation of immune-associated genes was associated with inhibition of viral replication and progression of viral infection [26]. Similarly, in the present study, upregulation of immune genes and downregulation of certain metabolism-related genes were observed in the pancreatitis-associated DHAV-1 infection group compared with the control group. However, significant KEGG enrichment was observed in the pancreatitis-associated DHAV-1 infection group compared with the classical-type DHAV-1 infection group that was mainly involved metabolism, including the glycine, serine, and threonine metabolism pathways. These results suggest that differences in metabolism functions in the DHAV infection group may contribute to the different DHAV-1 phenotypes.

D-3-phosphoglycerate dehydrogenase (PHGDH), phosphoserine aminotransferase (PSAT), and phosphoserine phosphatase (PSP) are involved in three steps of serine biosynthesis [27]. Initially, 3-phosphoglycerate is converted to 3-phosphohydroxypyruvate by the enzyme PGDH. Subsequently, PSAT converts 3-phosphohydroxypyruvate to 3-phosphoserine. Finally, 3-phosphoserine is converted to L-serine by PSP [28]. L-serine is an important precursor involved in various processes, such as synthesis of proteins and phospholipids as well as the synthesis of tetrahydrofolate metabolites and specific amino acids, namely glycine, cysteine, and D-serine [29, 30]. Previous studies have shown that L-serine supplementation can inhibit alcoholic fatty liver formation in mice and rats [31]. In the present study, the expression levels of the PHGDH, PSAT, and PSP genes were significantly lower in pancreatitis-associated-DHAV-1-infected ducklings than in the classical-type DHAV-1 group, suggesting that serine metabolism disorders are involved in the pancreatitis-associated DHAV-1 infection.

L-serine dehydratase (SDH) catalyzes the deamination of L-serine to yield ammonia and pyruvate. This enzyme uses L-threonine as a substrate to yield 2-oxobutanoate, which is a part of the valine, leucine, and isoleucine biosynthetic pathways. The decrease in serine dehydratase levels suggests that the pathway of conversion of serine to pyruvate was impaired, further affecting the valine, leucine, and isoleucine biosynthetic pathways [32–34].

TLRs play a critical role in innate immune responses. In recent years, the role of innate immunity and its interaction with adaptive immunity have been extensively investigated. In the TLR pathway, TLR2 plays a critical role in the induction of innate and inflammatory responses [35, 36]. Although TLR2 recognizes various bacterial components, recent studies have indicated that TLR2 is triggered by the hepatitis C virus core protein and NS3, leading to the activation of inflammatory cells [37, 38]. In the present study, the expression levels of the TLR2 gene were 10.63- and 7.62-fold higher in the pancreatitis-associated DHAV-1 and classical-type DHAV-1 infection groups, respectively, than in the control group, indicating that TLR2 may be involved in the host response to DHAV infection. TLR4 and CD14 have recently been shown to be major lipopolysaccharide (LPS) receptors. Mutations in mouse and human TLR4 were found to be associated with hyporesponsiveness to LPS and to confer an increased risk of infection with Gram-negative bacteria [39–41]. In addition to its interaction with LPS, the TLR4/CD14 complex interacts with viruses and proteins, such as the respiratory syncytial virus and fibrinogen [42–44]. In the present study, the TLR4/CD14 expression levels in the pancreatitis-associated DHAV-1 and classical-type DHAV-1 infection groups were much higher than in uninfected ducklings, indicating that TLR4/CD14 may be involved in the host response to pancreatitis-associated DHAV-1 and classical-type DHAV-1 infection. Surprisingly, TLR4 expression was significantly higher in the pancreatitis-associated DHAV-1 and classical-type DHAV-1 groups than in the mock-infected group, with an increase of 51.63- and 38.59-fold, respectively. TLR7 can detect single-stranded RNA (ssRNA) molecules and induce pro-inflammatory factors, such as the type I interferon, to stimulate the body's nonspecific immune response. Activation of TLR7 initiates downstream signaling cascades via induction of transcription factors such as IRF7. This induces the production of pro-inflammatory cytokines and chemokines that are involved in various viral infection outcomes, including spontaneous clearance and viral persistence [45]. In the present study, upregulation of TLR7, IRF7, and IFN-β expression was observed in the pancreatitis-associated DHAV-1 and classical-type DHAV-1 groups, which is consistent with the findings of previous studies [46, 47].

In summary, transcriptome analysis of pancreatic tissues derived from classical-type DHAV-1- and/or pancreatitis-associated-DHAV-1-infected ducks was performed. Infection with pancreatitis-associated DHAV-1 caused yellowing and hemorrhagic lesions in the pancreatic tissues of ducklings and was associated with differences in the expression levels of D-3-phosphoglyceratedehydrogenase, phosphoserine aminotransferase, and phosphoserine phosphatase, which are involved in the glycine, serine, and threonine metabolism pathways. These genes were significantly downregulated in the pancreatitis-associated-DHAV-1-infected group compared with the classical-type-DHAV-1-infected group, indicating that intensive metabolism disorders may contribute to the different phenotypes of DHAV-1 infection.

## Data Availability

The raw data have been deposited in the NCBI database, with [classical type DHAV-1-infected group accession number: SRR7239978 and SRR7239979], [Control group accession number: SRR7239984 and SRR7239985], and [pancreatitis-associated DHAV-1-infected group accession number: SRR7239988 and SRR7239989], [https://www.ncbi.nlm.nih.gov/sra/?term=SRR7239978, https://www.ncbi.nlm.nih.gov/sra/?term=SRR7239979, https://www.ncbi.nlm.nih.gov/sra/?term=SRR7239984, https://www.ncbi.nlm.nih.gov/sra/?term=SRR7239985, https://www.ncbi.nlm.nih.gov/sra/?term=SRR7239988, https://www.ncbi.nlm.nih.gov/sra/?term=SRR7239989]

## Abbreviations

KEGG: Kyoto Encyclopedia of Genes and Genomes
GO: Gene Ontology
RT-PCR: Reverse transcription polymerase chain reaction
ELISA: Enzyme-linked immunosorbent assay
DEG: Differentially expressed gene
BLAST: Basic Local Alignment Search Tool
ORF: Open reading frame
